# Bifunctional acid–base mesoporous silica@aqueous miscible organic-layered double hydroxides†

**DOI:** 10.1039/c9ra00188c

**Published:** 2019-01-28

**Authors:** Hongri Suo, Haohong Duan, Chunping Chen, Jean-Charles Buffet, Dermot O'Hare

**Affiliations:** Chemistry Research Laboratory, Department of Chemistry, University of Oxford 12 Mansfield Road Oxford OX1 3TA UK dermot.ohare@chem.ox.ac.uk +44 (0)1865 272686

## Abstract

A facile method for the synthesis of a series of mesoporous silica nanoporous (MSN) aqueous miscible organic layered double hydroxide core@shell nanocomposites using MCM-41, Al-MCM-41, SBA-15, and MCM-48 as the core is reported. These materials exhibit hierarchical morphologies with high surface areas and good porosity. Chemically, these materials offer controllable bifunctional basicity and acidity.

## Abstract 2

Core@shell materials which exhibit hierarchical morphology with ultra high surface area and controllable pore size and structure have been synthesised.

## Graphical abstract

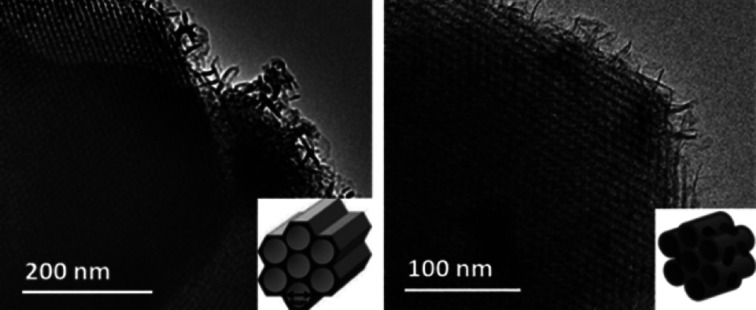

## Introduction

Layered double hydroxides (LDH) have been widely studied and used as catalysts, catalyst supports and absorbents^1–6^ owing to their inherent cation and anion substitutional flexibility. However, LDH particles synthesised by traditional methods (*e.g.* co-precipitation) typically exhibit platelet morphologies that have low BET surface areas due to the *ab*-face stacking aggregation of the primary LDH plates. Growing LDH nanosheets vertically on a core support is an effective strategy to overcome this inherent issue. Using different core materials enables the composite to gain additional features such as electronic, optical and magnetic functions. Ideally, we are hoping to observe synergistic chemical or physical properties.^7,8^

Since the successful synthesis of Mobil Composition of Matter no. 41 (MCM-41) in 1992,^9^ MCM-41 has been regarded as an important class of crystalline mesoporous silicon-based material. The framework of which is constructed of corner-sharing TO_4_ tetrahedral (where T represents a tetrahedrally coordinated Si, Al, or a heteroatom). Mesoporous silicas have been applied to catalysis, adsorption, separation, sensing, drug delivering devices, biodiesel production and nanotechnology due to their well-organised, template-directed porosity with high surface areas, large pore volumes, and tailorable pore size (2–50 nm).^10–14^ To date, there have been very limited effort devoted to the synthesis of core–shell LDH based materials using mesoporous silica nanoparticle (MSN) used as the core and LDH used as the shell. Mesoporous silica@CoAl-LDH spheres was synthesised using a layer-by-layer assembly method.^15^

Herein, we report a facile general method to synthesise mesoporous silica@AMO-Mg_3_Al-CO_3_-LDHs. The method has been exemplified using a range of different types of mesoporous silica nanoparticle (MSN), MCM-41, Al-MCM-41, SBA-15, and MCM-48. The LDH can be grown *in situ* on the surface of mesoporous silica at room temperature without any pre-treatment. We found that the rate of addition of the Mg and Al salts (3 : 1 ratio) to the MSN slurry was key to getting efficient growth rather than nucleation of a separate bulk LDH impurity phase. After ageing and dispersing, an aqueous miscible organic solvent treatment (AMOST) method was carried out before isolation. The AMOST method is an efficient and simple synthetic method, developed by O'Hare and co-workers.^16–19^ It reduces the hydrogen bonding network within the primary LDH particles, thus inhibiting aggregation of primary LDH platelets.

## Results and discussion

**Fig. 1:**
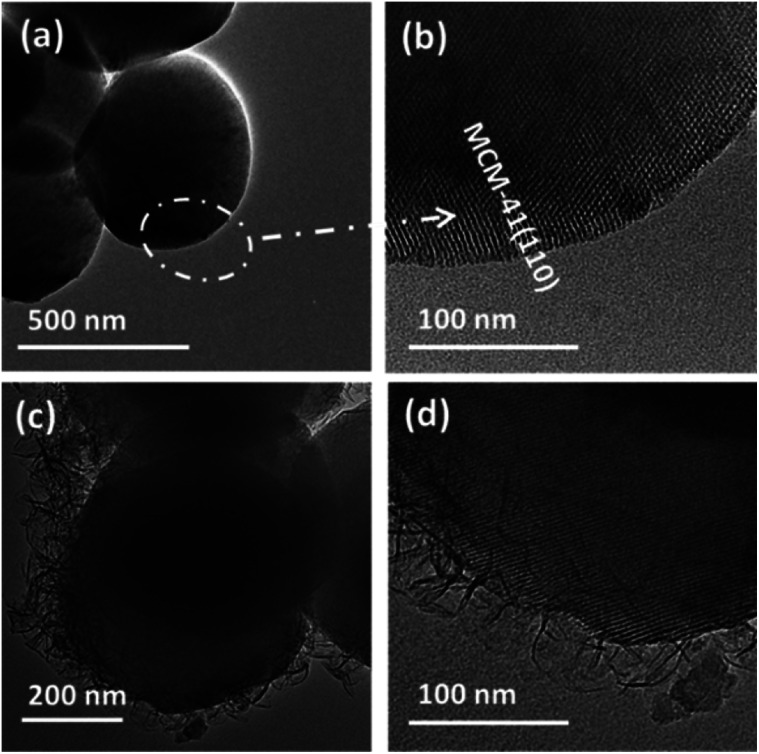
HRTEM images of (a and b) MCM-41 spheres and (c and d) MCM-41@AMO-Mg_3_Al-CO_3_-LDH (synthesis conditions: pH 9.5, room temperature, Mg/Al 3 : 1).

High resolution transmission electron microscopy (HRTEM) images of MCM-41 and MCM-41@AMO-Mg_3_Al-CO_3_-LDH are shown in Fig. 1. Fig. 1a and b show that the as-synthesised MCM-41 is composed of monodispersed nanospheres with uniform particle size of around 600 nm, the (110) lattice fringes are estimated to be 3.3 nm. Fig. 1c and d, show the HRTEM images of the sample following *in situ* co-precipitation of Mg_3_Al-CO_3_ LDH in the presence of MCM-41 and then AMOST treatment. The Mg_3_Al-CO_3_-LDH nanosheets have vertically and randomly grown on the MCM-41 surface to form a hierarchical structure. The thickness of the AMO-Mg_3_Al-CO_3_-LDH shell is about 50–100 nm. Lattice fringes arising from the core MCM-41 (Fig. 1d) can be observed beneath the AMO-Mg_3_Al-CO_3_-LDH shell.

**Fig. 2:**
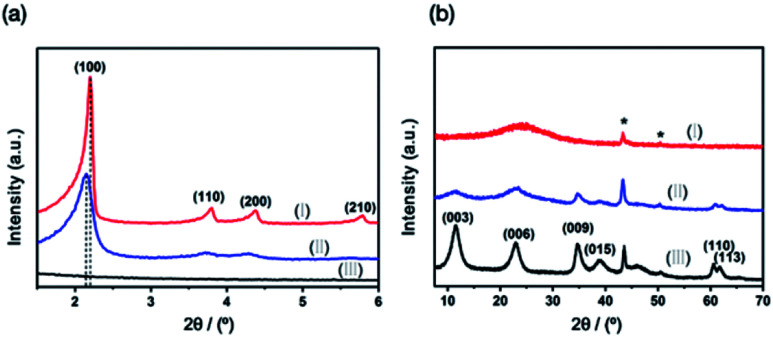
(a) Low angle and (b) wide angle XRD pattern of (I) MCM-41, (II) MCM-41@AMO-Mg_3_Al-CO_3_-LDH and (III) AMO-Mg_3_Al-CO_3_-LDH; * is the signal due to the sample holder.

The crystalline phase composition and phase crystallinity of MCM-41@AMO-Mg_3_Al-LDH was studied using X-ray powder diffraction (XRD) as shown in Fig. 2. The four distinct Bragg reflections of MCM-41 are observed in low angle XRD pattern (Fig. 2a), these reflections can be indexed as (100), (110), (200) and (210), and are associated with the two-dimensional hexagonal mesoporous structure (space group *P*6*m*).^20^ As MCM-41 consists of an amorphous silica framework, only a broad Bragg scattering feature around 2*θ* = 23° is observed in the wide angle XRD pattern (Fig. 2b). The AMO-Mg_3_Al-CO_3_-LDH shell exhibits a typical LDH structure with rhombohedral symmetry as shown in Fig. 2b.

The XRD of MCM-41@AMO-Mg_3_Al-CO_3_-LDH was found to be a weighted sum of the XRDs of MCM-41 and AMO-Mg_3_Al-CO_3_-LDH. We could not detect any perturbation in the lattice constants of the LDH shell. However, a slight peak shift of the Bragg reflection (100) from the MCM-41 component indicates the *d*-spacing of the core in MCM-41@AMO-Mg_3_Al-CO_3_-LDH is larger than that of a pristine MCM-41. We believe this may be due to desilication and incorporation of Al into the silica framework.^21,22^

The thermal properties and phase composition were evaluated using thermal gravimetric analysis (TGA). As shown in Fig. S1,† MCM-41@AMO-Mg_3_Al-LDH exhibits typical weight losses of LDH in the range of 30–800 °C. AMO-LDH phase fraction was estimated to be about 54 wt% of the core@shell material.

**Fig. 3:**
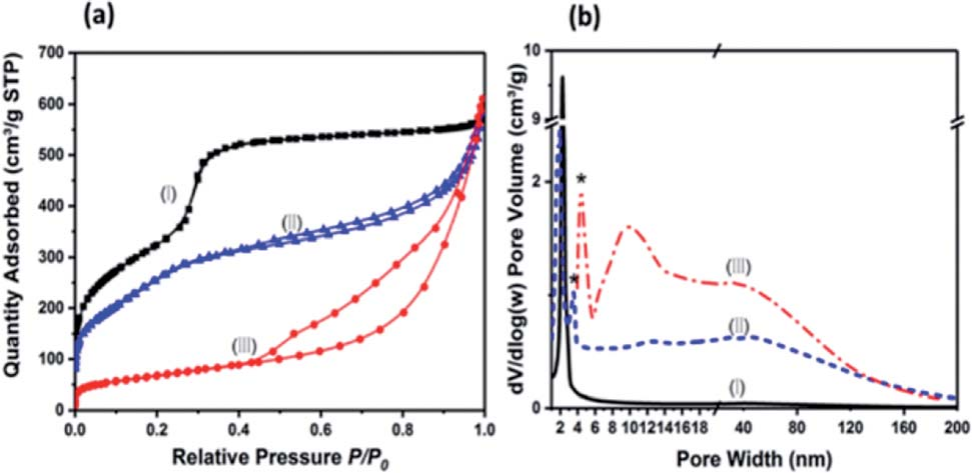
(a) N_2_ adsorption–desorption isotherms and (b) pore size distribution of (I) MCM-41, (II) MCM-41@AMO-Mg_3_Al-CO_3_-LDH and (III) AMO-Mg_3_Al-CO_3_-LDH; * is the tensile strength effect.

The N_2_ Brunauer–Emmett–Teller (BET) adsorption/desorption isotherms and pore size distribution are shown in the Fig. 3(a and b). The isotherm for MCM-41 exhibits a IV response with a capillary condensation step at relative pressure (*P*/*P*_0_) of 0.3–0.4, which is a characteristic isotherm of mesoporous materials according to IUPAC classification.^23^AMO-Mg_3_Al-CO_3_-LDH shows a typical IV isotherm with a H3 type hysteresis loop starting at *P*/*P*_0_ = 0.42, which suggests it is composed of plate-like materials with a slit-shape pore structure. This behaviour is consistent with our previous reports.^17^ The N_2_ adsorption/desorption isotherm for MCM-41@AMO-Mg_3_Al-CO_3_-LDH shows a combination of the capillary condensation and hysteresis loop, indicating that it has both mesoporosity and macroporosity. The measured high specific surface area of 897 m^2^ g^−1^ and large pore volume of 0.91 cm^3^ g^−1^ can be mainly attributed to high porosity of the core material (MCM-41). The calculated pore size distribution as shown in Fig. 3b. The core material (MCM-41) presents a narrow pore size distribution around 1.9 nm. While AMO-Mg_3_Al-CO_3_-LDH shows multipores in the range of 4–200 nm; MCM-41@AMO-Mg_3_Al-CO_3_-LDH consists of both small pore size with narrow pore distribution (from MCM-41) and hierarchical porosity range of 10–200 nm (mainly from AMO-Mg_3_Al-CO_3_-LDH shell). Compared to the pristine MCM-41 material, the shrunk pore size of MCM-41@AMO-Mg_3_Al-CO_3_-LDH may be due to the wet grafting on the internal surface of the mesoporous channel by –OH groups, or exchange of Na^+^, Mg^2+^ or Al^3+^ species into framework channels.^21^

**Fig. 4:**
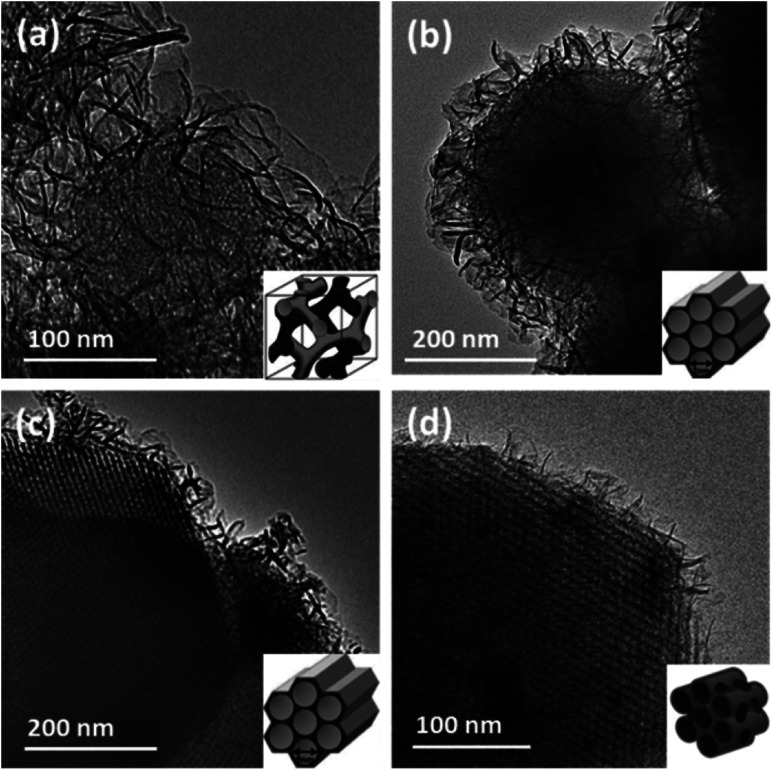
TEM images of MSN@AMO-LDH using different MSN as the core materials (a) MCM-48@AMO-Mg_3_Al-CO_3_-LDH, (b) Al-MCM-41@AMO-Mg_3_Al-CO_3_-LDH, (c) SBA-15@AMO-Mg_3_Al-CO_3_-LDH and (d) P-SBA-15@AMO-Mg_3_Al-CO_3_-LDH (inset images are corresponding to 3D schematic diagrams of porous structure). Synthesis conditions: pH 9.5, room temperature, Mg/Al 3 : 1.

The synthesis approach was extended to prepare a various range of core–shell MSN@AMO-Mg_3_Al-CO_3_-LDH composites using Al-MCM-41, SBA-15, MCM-48 and P-SBA-150. The XRD patterns confirmed that the core–shell materials consist of both LDH and core materials (Fig. S2 and S3†). A summary of the morphologies of these core–shell MSN@AMO-Mg_3_Al-CO_3_-LDH composites is shown in Fig. 4 (together with a 3D schematic diagram of their pore structures, inset of Fig. 4). We found that the AMO-Mg_3_Al-CO_3_-LDH nanosheets grow vertically on the MSN core materials with a hierarchical structure. The thickness and coverage of AMO-Mg_3_Al-CO_3_-LDH shell varies according to the different pore structures and surface chemistry of the core materials.

The mesoporous fringes with different widths can be clearly observed under the AMO-Mg_3_Al-CO_3_-LDH shell. This is consistent with the results from low angle XRD (Fig. S2†). The phase ratio between MSN and AMO-Mg_3_Al-CO_3_-LDH can be controlled by varying the size of MSN particles and the quantity of metal salt feed solutions precipitating the LDH (Fig. S4†). N_2_ BET adsorption measurements were carried out with MSN@AMO-Mg_3_Al-CO_3_-LDHs. A summary of specific surface areas of the MSN@AMO-Mg_3_Al-CO_3_-LDHs are shown in Table 1 (605–1005 m^2^ g^−1^). The isotherm and pore size distribution are shown Fig. S5.† Although MCM-48@AMO-Mg_3_Al-CO_3_-LDH has a similar pore size (2 nm) as MCM-41@AMO-Mg_3_Al-CO_3_-LDH, the XRD pattern of MCM-48 in Fig. S2† indicated that its actual pore structure is a three dimensional cubic, 3D schematic diagram of which is shown in the inset of Fig. 4a. SBA-15@AMO-Mg_3_Al-CO_3_-LDH has the same hexagonal channel as MCM-41@AMO-Mg_3_Al-CO_3_-LDH but a larger pore size of 6 nm. Using a P-SBA-15 core introduced two different pores of 1 and 8 nm into the new composite. Compared with SBA-15, the additional pore at 1 nm was formed by the PVA template as highlighted in the pore size distributions of SBA-15-PVA@AMO-Mg_3_Al-CO_3_-LDH obtained by NLDFT method (inset of Fig. S5(II)†). This connects the larger mesopores (8 nm) and provides better transport channels through the material. In practical applications, the pore structure and shape selectivity of catalyst, support, or absorbent play a very important role in a wide range of industrial processes.^24–26^ The porosity control is a great advantage of the MSN@AMO-Mg_3_Al-CO_3_-LDH.

**Table 1:** Summary of the N_2_ BET specific surface areas and pore volumes of MSN@AMO-Mg_3_Al-CO_3_-LDH using different MSN nanoparticles as the core materials

Samples	Surface area (m^2^ g^−1^)	Pore volume (cm^3^ g^−1^)
MCM-41	1160	0.88
MCM-41@AMO-Mg_3_AlCO_3_-LDH	897	0.91
MCM-41@AMO-Mg_3_AlCO_3_-LDH-10.5	724	0.94
SBA-15	867	1.21
SBA-15@AMO-Mg_3_AlCO_3_-LDH	668	1.20
MCM-48@AMO-Mg_3_AlCO_3_-LDH	1005	1.17
SBA-15-PVA@AMO-Mg_3_AlCO_3_-LDH	605	1.24
Al-MCM-41	838	0.82
Al-MCM-41@AMO-Mg_3_AlCO_3_-LDH-10.5	796	1.55
AMO-Mg_3_AlCO_3_-LDH	200	0.90

**Fig. 5:**
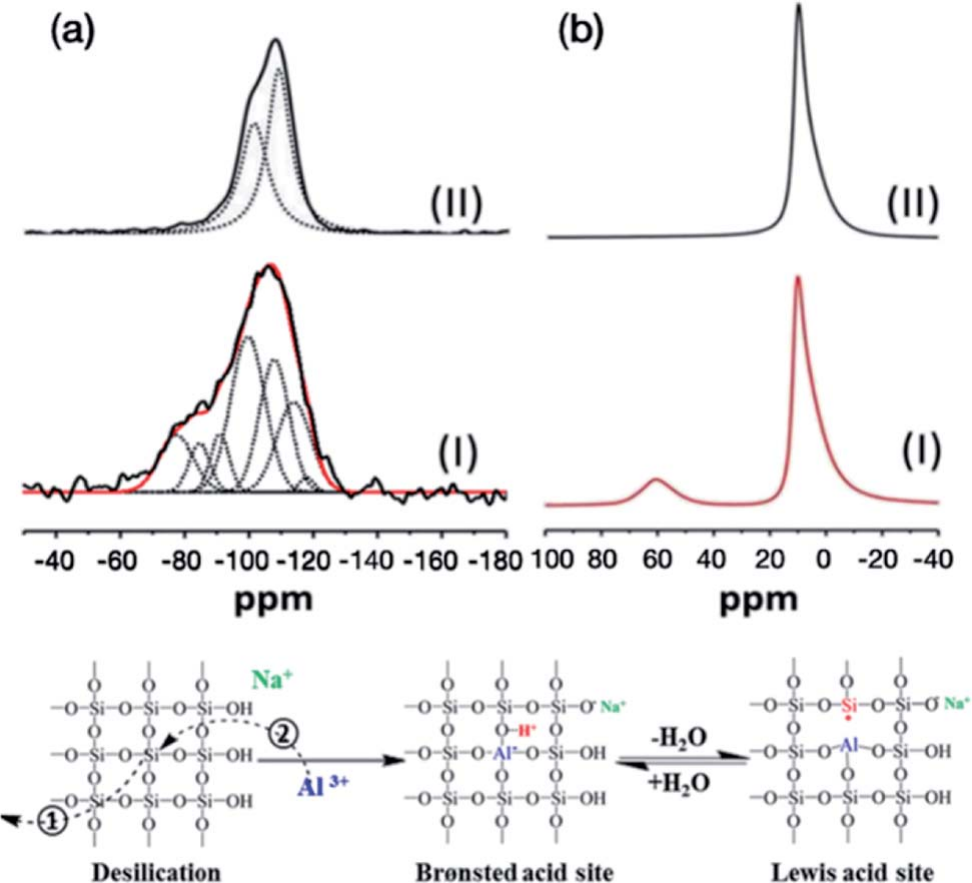
Solid state NMR spectra of (a) ^29^Si NMR (I) MCM-41@AMO-Mg_3_Al-CO_3_-LDH and (II) MCM-41; (b) ^27^Al NMR (I) MCM-41@AMO-Mg_3_Al-CO_3_-LDH and (II) AMO-Mg_3_Al-CO_3_-LDH.

^29^Si and ^27^Al solid state NMR (SSNMR) spectra (Fig. 5) have been recorded in order to investigate the silicon and aluminium environments before and after nucleating and growing AMO-Mg_3_Al-CO_3_-LDH on the surface of MCM-41. In agreement with previous studies, the ^29^Si SSNMR of MCM-41 can be deconvoluted into two resonances centred at −101 and −110 ppm which correspond to Q3(SiO)_3_SiOH and Q4(SiO)_4_Si species respectively.^27–29^ However, the signal of MCM-41@AMO-Mg_3_Al-LDH (Fig. 5aI) can be deconvoluted into more than two resonances as shown in the Table S1.† The lower field resonances at −100 and −108 ppm are probably due to the effect of cation exchange between sodium or magnesium and protons during the synthesis of AMO-Mg_3_Al-CO_3_-LDH shell.^29^ The three extra broad peaks at −91, −85, and −77 ppm are attributed to the combination of Q2(SiO)_2_Si(OH)_2_, Q3(SiO)_2_(AlO)SiOH, Q4Si(3Al), Q4Si(2Al), Q4Si(2Al) or Q4Si(1Al).^30–32^ The resonances at 113 and 117 ppm can be assigned to the cristobalite Q4 Si(0Al) which is an outcome of the crystallographic rearrangement of the silica mesoporous framework of MCM-41 materials. In the solid state ^27^Al MAS NMR, the resonance at 10 ppm can be attributed to octahedral Al species from the LDH structure.^33^ However, an additional resonance at 60 ppm was found in MCM-41@AMO-Mg_3_Al-CO_3_-LDH samples. This resonance can be assigned to 4-coordinated Al (including AlO_4_ species bonded to Si). After the desilication, besides the rearrangement of O–Si–O (Fig. 5 Step①), some of the vacancies can be refilled by aluminium ions during coprecipitation (Fig. 5 Step②). This is similar to the method commonly used to modify pure mesoporous silicas and to enhance their ion exchange capacity and acidity by using Al(NO_3_)_3_·9H_2_O or AlCl_3_ solution as aluminium source.^34,35^ By replacing silicon by aluminium, new Brønsted acidity site can be formed on the MCM-41 framework. Brønsted acid can easily interconvert with Lewis acid site through dehydration and hydration (Fig. 5 right).

The acidity and basicity of the composite MCM-41@AMO-Mg_3_Al-CO_3_-LDH after calcination at 400 °C for 6 h (MCM-41@AMO-Mg_3_Al-CO_3_-LDO) was measured by NH_3_- and CO_2_-temperature programmed desorption (TPD) (Fig. S6†). The CO_2_-TPD of MCM-41@AMO-Mg_3_Al-CO_3_-LDO exhibits a strong peak at around 150 °C with a broad weak peak at around 270 °C, which are assigned to OH groups (weak basic sites) and metal–oxygen pairs (moderate basic sites), respectively.^36,37^ The NH_3_-TPD of MCM-41@AMO-Mg_3_Al-CO_3_-LDO also shows overlapping peaks at 160 and 250 °C, which are attributed to weak and moderate acid sites, respectively (Table S2†). AMO-Mg_3_Al-CO_3_-LDO exhibits weak-moderate basic strength with a total basic number of 0.4993 mmol g^−1^. MCM-41 is a well-known acid material without basic sites. However, after growing the AMO-Mg_3_Al-CO_3_-LDO shell, both weak and moderate basic sites were found (0.2875 mmol g^−1^).

The total acidity for MCM-41 was found to *ca*. 0.0184 mmol g^−1^ while AMO-Mg_3_Al-CO_3_-LDO did not show any acidity. The core–shell MCM-41@AMO-Mg_3_Al-CO_3_-LDO exhibits a higher total acidity of 0.3380 mmol g^−1^ compared to pristine MCM-41. This could be due to the alumination of MCM-41 after desilication during the core–shell synthesis (Fig. 5 Step②). These findings suggest that both acidity and basicity can be introduced into core–shell composites material simultaneously by growing LDH shell on MSN materials.

## Conclusion

We have developed a versatile method for the synthesis of a series of MSN@AMO-Mg_3_Al-CO_3_-LDH composite materials with a hierarchical structure. The obtained MSN@AMO-Mg_3_Al-CO_3_-LDH exhibit high surface areas. The pore size and structure of the core–shell materials can be controlled by changing the core material.

The obtained core–shell composites not only combine the properties from the parent materials (LDH and MSNs) but also introduces new properties (such as higher amount of acid sites). TPD analysis shows that MCM-41@AMO-Mg_3_Al-CO_3_-LDH is a bifunctional acid–base material with basic activity site derived from the AMO-Mg_3_Al-CO_3_-LDH shell and acidity from the core (partially substituted by Al).

MSN@AMO-Mg_3_Al-CO_3_-LDH with their adjustable pore structure, pore size and acid/base properties should provide exciting prospects as absorbents, catalysts or catalyst supports.

## Experimental part

### General details

Transmission electron microscopy (TEM) analyses were performed on a JEOL 2100 microscope with an accelerating voltage of 200 kV.

Powder X-ray diffraction (XRD) data were collected on a PANAnalytical X'Pert Pro diffractometer at 40 kV and 40 mA using Cu Kα radiation (*α*_1_ = 1.54057 Å, *α*_2_ = 1.54433 Å, weighted average = 1.54178 Å). The reflections at 2*θ* = 43–44° and 50° are produced by the XRD sample holder.

The solid state NMR spectroscopy (^29^Si and ^27^Al) were recorded on a Varian Chemagnetics CMX Infinity 200 (4.7 T). Samples were packed in 7.5 mm zirconia rotors. A double resonance MAS probe was used for all measurements and a MAS rate of 4 kHz for ^29^Si, whereas MAS rate of 6 kHz was used for ^27^Al. ^27^Al MAS NMR spectra were acquired with a single pulse excitation applied using a short pulse length (0.7 μs). Each spectrum resulted from 2000 scans separated by 1 s delay. The ^27^Al chemical shifts are referenced to an aqueous solution of Al(NO_3_)_3_ (*δ* = 0 ppm). In order to obtain the quantitative ^29^Si DPMAS NMR spectra, 5000 transients were typically acquired with an acquisition time of 68 ms (1024 data points zero filled to 16 K) and a recycle delay of 30 s. All ^29^Si spectra were externally referenced to kaolinite (taken to be at *δ* = −91.7 ppm on a scale where *δ*(TMS) = 0 ppm) as a secondary reference.

Thermogravimetric analysis (TGA) measurements were collected using a Netzsch STA 409 PC instrument. The sample (10–20 mg) was heated in a corundum crucible between 30 and 800 °C at a heating rate of 5 °C min^−1^ under a flowing stream of nitrogen.

Brunauer–Emmett–Teller (BET) specific surface areas were measured from the N_2_ adsorption and desorption isotherms at 77 K collected from a Micromeritic 3-Flex.

Temperature programmed desorption (TPD) was performed on Micromeritics AutoChem II 2920 using a flow of N_2_ (30 mL min^−1^) ramping from 30 to 400 °C at a rate of 5.0 °C min^−1^ for 6 hours, then cool down to 80 °C; CO_2_ or NH_3_ was feed in the reactor for 1 h at 80 °C, then flow N_2_ at 100 °C for half hour to remove the physical adsorption CO_2_ or ammonium, the TCD signal is recorded from 100–600 °C at the rate of 10 °C min^−1^.

### Synthesis of mesoporous silica nanoparticles

#### MCM-41/Al-MCM-41

MCM-41 and Al-MCM-41 were synthesised according to a published literature.^20^ Firstly, 26.7 g of ammonia solution (25% NH_4_OH, Merck) was mixed with 105 g of distilled water. Then, 0.5 g of cetyltrimethyl ammonium bromide (CTAB, Sigma-Aldrich) was added to the solution. For Al-MCM-41, 0.1 g of aluminium isopropoxide (Sigma-Aldrich) was added to a solution with a SiO_2_ : Al_2_O_3_ of 50 : 1. The mixture was placed in an oil bath under stirring and heated to a homogeneous solution at 75 °C. When a clear solution was obtained, 2.35 g of tetraethyl orthosilicate (TEOS, Sigma-Aldrich) was added dropwise. After 3 h of stirring and heating at 75 °C, the obtained colourless solid was washed with water and dry in a vacuum oven at room temperature, over-night. Finally, the solid was calcined at 550 °C for 6 h in air to remove the CTAB from the pores.

#### SBA-15/P-SBA-15

SBA-15 and P-SBA-15 were synthesised according to published literature.^38,39^ 4.0 g of triblock copolymer Pluronic (P123, Sigma-Aldrich) was added to a mixture of 30 g of water and 120 g of 2 M HCl (Sigma-Aldrich) aqueous solution in a Teflon-lined container (for the SBA-P-15 sample, 3 mL 1% polyvinyl alcohol (PVA, Sigma-Aldrich) was added in the solution). The mixture was stirred overnight at 35 °C. Then, 8.50 g of TEOS was added to this solution under vigorous stirring at 1000 rpm. After 5 minutes of stirring, the mixture was kept under static conditions at 35 °C for 20 h, followed by 24 h at 100 °C. The solid product was collected by filtration, washed with water, dried, and calcined at 550 °C in air.

#### MCM-48

A typical preparation of MCM-48-type MSN is as follows:^40^ 0.5 g of CTAB and 2.05 g of triblock copolymer F127 (Sigma-Aldrich) are dissolved in distilled water (96 mL), 34 g of EtOH (99.99%, Sigma-Aldrich), and 10.05 g of 29 wt% ammonium solution at room temperature. After complete dissolution, 1.8 g of TEOS is added into the mixture at once. After 1 minute stirring at 1000 rpm, the mixture was kept at static condition for 24 h at room temperature for further condensation. The colourless solid was recovered by ultra high speed centrifuge, washed with water, and dried at 75 °C in air. The final MCM-48 MSN materials were obtained after calcinations at 550 °C in air.

### Synthesis of MSN@AMO-Mg_3_Al-CO_3_-LDH

The MSN@AMO-Mg_3_Al-CO_3_-LDH particles were synthesised by co-precipitation method: mesoporous silica nanoparticles (0.2–0.5 g) were dispersed in deionised water (20 mL) using an ultrasound treatment for 30 minutes. Then, the anion salt Na_2_CO_3_ (0.96 mmol, Sigma-Aldrich) was added to the solution and further treated by ultrasound for 5 minutes; the final solution was named A. Then, the metal precursor solution (19.2 mL) containing Mg(NO_3_)_2_·6H_2_O (1.08 mmol, Sigma-Aldrich) and Al(NO_3_)_3_·9H_2_O (0.36 mmol, Sigma-Aldrich) was added to solution A, at a rate of 60 mL h^−1^ with vigorous stirring. The pH of the solution was controlled at 9.5 by dropwise addition of 1 M NaOH. After ageing for 30 minutes with stirring at room temperature, the obtained solid was collected after centrifugation (5000 rpm for 5 minutes) and then re-dispersed in deionised water (40 mL) and stir for 1 h, the washing step was repeated twice. The final pH was around 7.

Aqueous Miscible Organic Solvent Treatment (AMOST or AMO) method^17^ is an efficient way to increase the surface area of typical pure LDH. AMO-method was used on the MSN@AMO-Mg_3_Al-CO_3_-LDH. Before final isolation, the solid was washed with ethanol (40 mL) once and disperse in ethanol (40 mL) again, then left to stir overnight at room temperature. The suspension was centrifuged and dried in vacuum oven at room temperature. Finally, the MSN@AMO-Mg_3_Al-CO_3_-LDH was obtained as a fluffy white power.

## Conflicts of interest

There are no conflicts to declare.

## Supplementary Material

RA-009-C9RA00188C-s001
