# Supplementary material for: Bifunctional acid–base mesoporous silica@aqueous miscible organic-layered double hydroxides
Source: RSC Adv. 2019 Jan 28;9(7):3749–54. doi: 10.1039/c9ra00188c (PMC9060557; doi:10.1039/c9ra00188c)
Supplement: RA-009-C9RA00188C-s001 [file RA-009-C9RA00188C-s001.pdf]

Electronic Supplementary Material (ESI) for RSC Advances.
This journal is © The Royal Society of Chemistry 2019

Electronic Supplementary material (ESI)

Bifunctional acid-base mesoporous silica@aqueous miscible organic-layered double hydroxides

Hongri Suo, Haohong Duan, Chunping Chen, Jean-Charles Buffet and Dermot O'Hare

Chemistry Research Laboratory, Department of Chemistry, University of Oxford,

12 Mansfield Road, Oxford, OX1 3TA, UK.

E-mail: Dermot.ohare@chem.ox.ac.uk

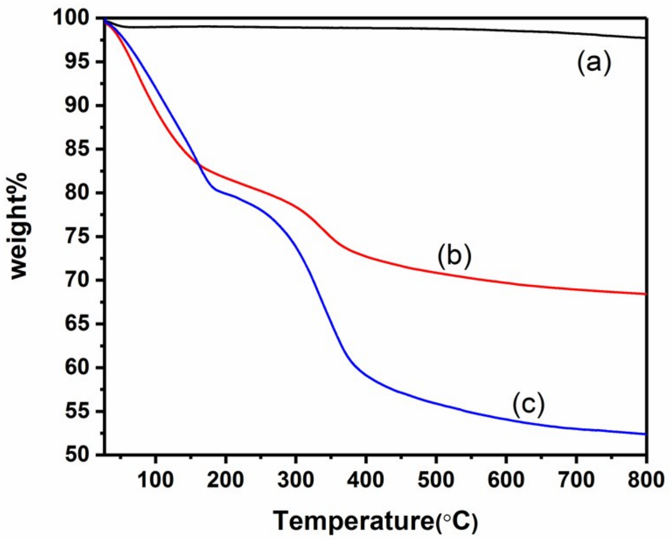

Fig. S1. TGA of (a) MCM-41, (b) MCM-41@AMO-Mg_3Al-CO_3-LDH and (c) AMO-Mg_3Al-CO_3-LDH (in the range of 30–800 °C).

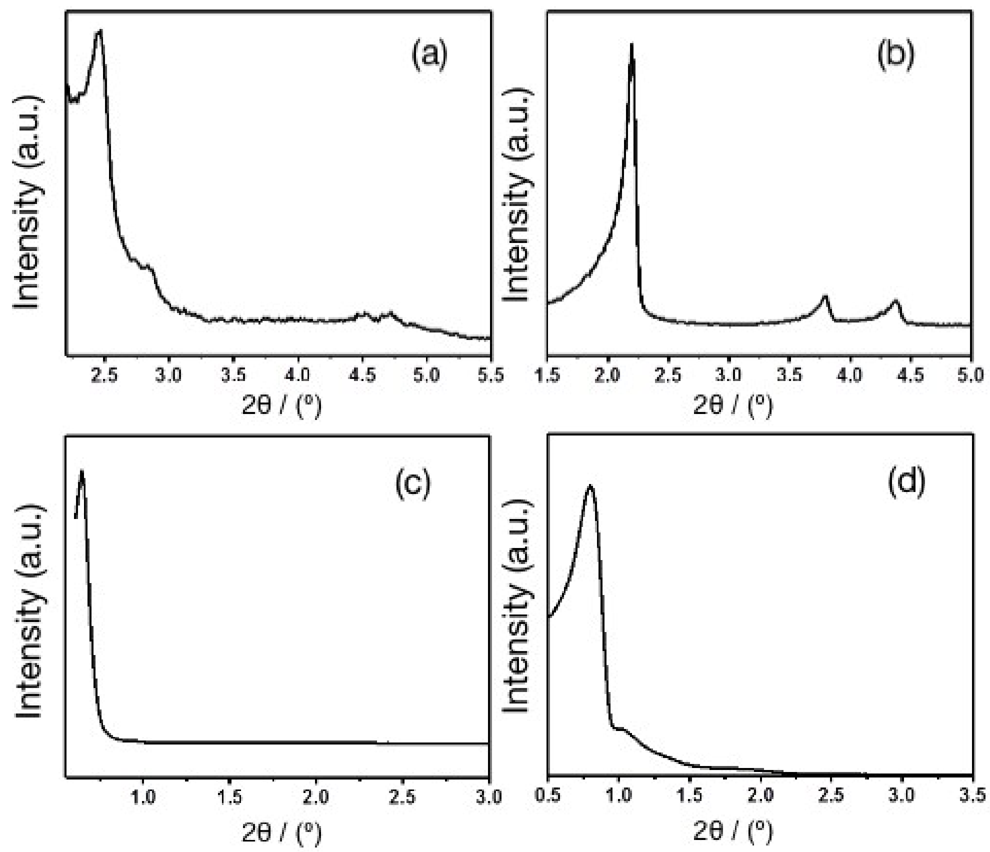

Fig. S2. Low angle XRD patterns for different MSN nanoparticles as the core materials: (a) MCM-48@AMO-Mg_3Al-CO_3-LDH, (b) MCM-41@AMO-Mg_3Al-CO_3-LDH, (c) SBA-15@AMO-Mg_3Al-CO_3-LDH and (d) P-SBA-15@AMO-Mg_3Al-CO_3-LDH.

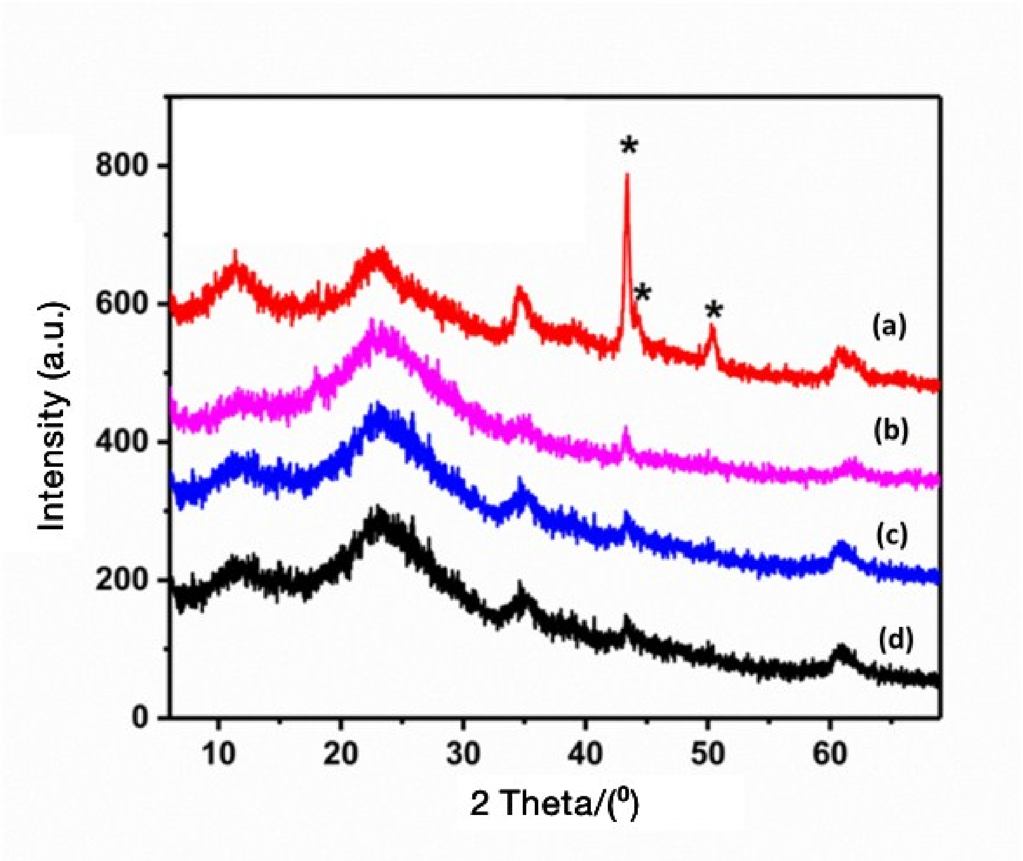

Fig. S3. High angle XRD patterns for different MSN nanoparticles as the core materials: (a) MCM-48@AMO-Mg_3Al-CO_3-LDH, (b) MCM-41@AMO-Mg_3Al-CO_3-LDH, (c) SBA-15@AMO-Mg_3Al-CO_3-LDH and (d) P-SBA-15@AMO-Mg_3Al-CO_3-LDH (* is the signal of sample holder).

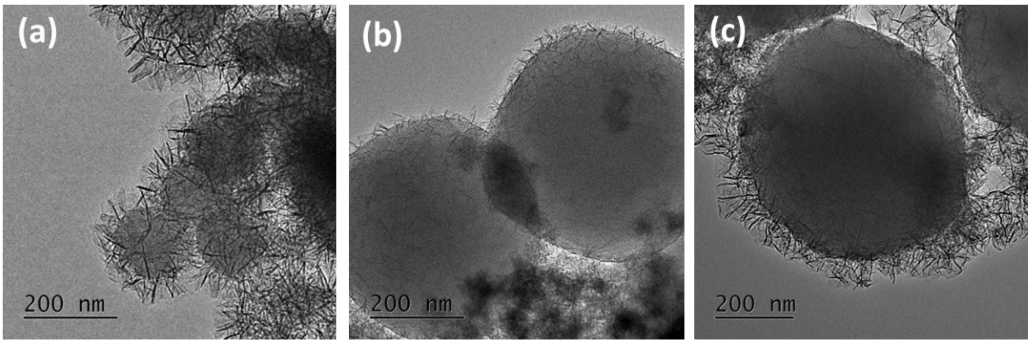

Fig. S4. TEM image of MSN@AMO-MgAl-CO_3-LDH with different diameter core and different Mg/Al (a) 150 nm-MCM-41@AMO-Mg_3Al-CO_3-LDH (70% AMO-LDH), (b) 600 nm-MCM-41@AMO-Mg_2Al-CO_3-LDH (54% AMO-LDH) and (c) 600 nm-MCM-41@AMO-Mg_3Al-CO_3-LDH (63% AMO-LDH). The weight percentage of AMO-LDH was calculated from TGA data.

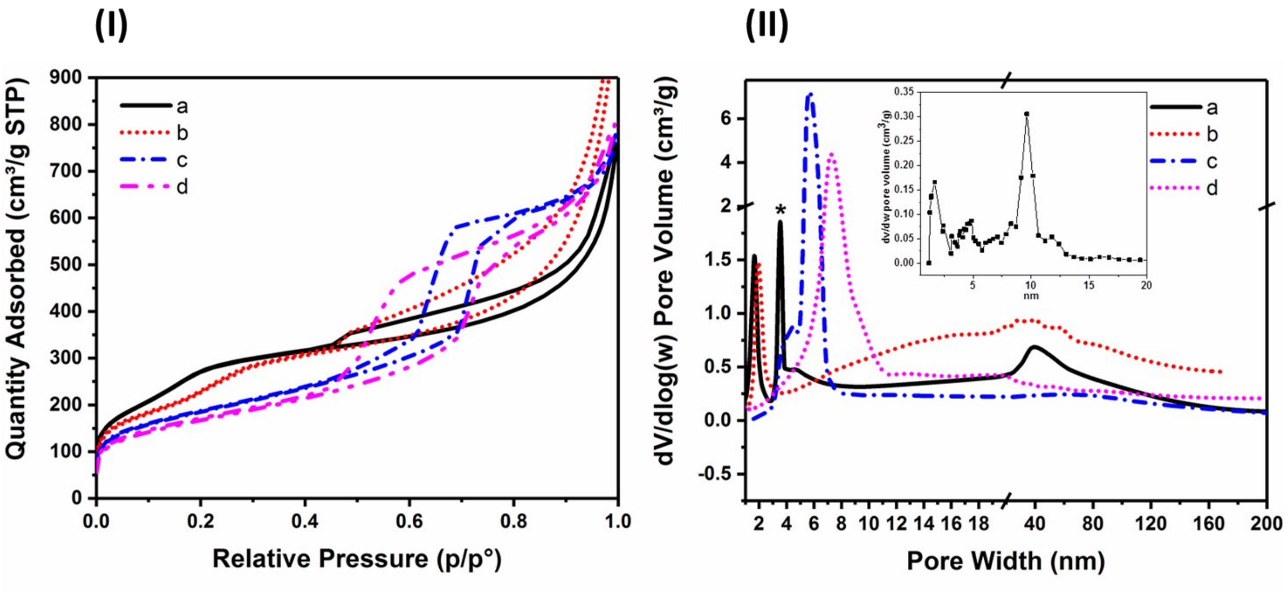

Fig. S5. (I) N_2 adsorption-desorption isotherm and (II) BJH pore size distribution of MSN@AMO-Mg_3Al-CO_3-LDH by using different MSN nanoparticles as the core materials: (a) MCM-48@AMO-Mg_3Al-CO_3-LDH, (b) MCM-41@AMO-Mg_3Al-CO_3-LDH, (c) SBA-15@AMO-Mg_3Al-CO_3-LDH and (d) P-SBA-15@AMO-Mg_3Al-CO_3-LDH. The insert picture is the pore size distributions of SBA-15-PVA@AMO-Mg_3Al-LDH obtained by NLDFT method and the * is the tensile strength effect.

Table S1. Silicon environment in MCM-41 and MCM-41@AMO-Mg_3Al-CO_3-LDH determined by ^{29}Si solid state NMR spectroscopy.

**MCM-41**	**δ (ppm)**	**Assignment**	**Percentage (%)**
1	-101	Q3 (SiO)3SiOH	68
2	-110	Q4 (SiO)4Si	32
**MCM41@ AMO-Mg_3Al-CO_3-LDH**	**δ (ppm)**	**Assignment**	**Percentage (%)**
1	-77.5	Q2 (SiO)2Si(OH)2, Q3(SiO)2(AlO)SiOH, Q4Si(3Al), Q4Si(2Al), Q4Si(1Al)	11.8
2	-85		6.2
3	-91		6.5
4	-100	Q3 (SiO)3SiOH	32
5	-108	Q4 (SiO)4Si	23
6	-113.8	Cristobalite	18.5
7	-117.8	Cristobalite	1.3

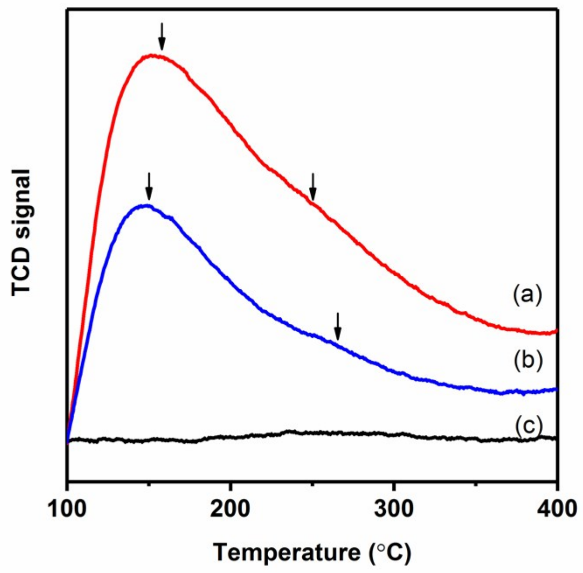

Fig S6. NH_3 TPD of (a) MCM-41, (b) MCM-41@AMO-Mg_3Al-CO_3-LDH and (c) CO_2 TPD of MCM-41@AMO-Mg_3Al-CO_3-LDH (in the range of 100 – 400 °C).

Table S2. Acidity / basicity quantitative evaluation of MCM-41, AMO-Mg_3Al-CO_3-LDH and composite MCM-41@AMO-Mg_3Al-CO_3-LDH by CO_2/NH_3-Temperature programmed desorption (TPD) measurement.

Acidity	Acid site	Amount (mmol/g)
MCM-41	weak	0.0184
AMO-Mg_3Al-CO_3-LDH (Mg/Al=3:1)	-	0.00 (after calibration)
MCM-41@AMO-Mg_3Al-CO_3-LDH	weak+moderate	0.3380 (after calibration)
**Alkalinity**	**Basic site**	**Amount (mmol/g)**
MCM-41	-	0.006(after calibration)
AMO-Mg_3Al-CO_3-LDH (Mg/Al=3:1)	weak+moderate	0.4993 (after calibration)
MCM-41@AMO-Mg_3Al-CO_3-LDH	weak+moderate	0.2875 (after calibration)
